# Anterior Chamber Paracentesis Offers a Less Painful Experience During Intravitreal Anti-vascular Endothelial Growth Factor Administration: An Intraindividual Study

**DOI:** 10.7759/cureus.20051

**Published:** 2021-11-30

**Authors:** Refika Hande Karakahya

**Affiliations:** 1 Ophthalmology, Lösante Hospital, Ankara, TUR

**Keywords:** anti-vegf, visual analogue scale, pain, intravitreal injection, anterior chamber paracentesis

## Abstract

Introduction: In order to improve comfort and compliance to treatment of the patient during the intravitreal injections (IVIs), relieving pain may help and provide getting better results. The purpose of the study was to evaluate the efficacy of anterior chamber paracentesis on pain perception and the factors related to pain perception during intravitreal injection procedures.

Material and methods: This prospective randomized study includes 212 eyes of 106 patients scheduled for bilateral IVI of ranibizumab 0.5 mg/0.05 cc under topical anesthesia. All patients underwent full ophthalmologic examination, including intraocular pressure (IOP), anterior chamber depth (ACD), and axial length (AL) measurements. Group 1 received IVI following anterior chamber paracentesis (ACP) and group 2 received IVI without ACP. Intraocular pressure was measured five minutes and 30 minutes after the procedure. Pain perception was assessed by visual analogue scale (VAS) grading from 0 to 10.

Results: Mean VAS score for groups 1 and 2 was recorded as 0.51±1.00 and 1.32±1.50, respectively. Correlation analysis revealed a positive correlation between VAS score and history of previous IVI, preinjection IOP values, and an inverse correlation with the presence of reflux in both groups, in addition to inverse correlation with ACD in group 2.

Conclusions: ACP may offer a comfortable, effective, and less painful alternative to prevent the acute rise in IOP after IVI, especially in patients with small anterior chambers, small vitreous volumes, with a history of multiple injections, and in patients with advanced glaucomatous optic neuropathy.

## Introduction

Intravitreal injections (IVI), maximizing the intraocular concentrations while minimizing the systemic exposure, has become the standard procedure in ophthalmologic conditions such as endophthalmitis, viral retinitis, age-related macular degeneration, cystoid macular edema, diabetic retinopathy, uveitis, retinal vascular occlusions, retinal detachment to deliver several therapeutics such as anti-infective and anti-inflammatory medications, immunomodulators, anticancer agents, gas and anti-vascular endothelial growth factor (anti-VEGF) [1].

After the introduction of anti-VEGF agents, IVI has become one of the most common procedures in ophthalmology in the treatment of conditions like exudative age-related macular degeneration, macular edema due to retinal vein occlusion, diabetic retinopathy, other types of choroidal neovascularization, vascular proliferative retinal diseases such as retinopathy of prematurity [2,3].

Nevertheless, the treatment protocols for anti-VEGF agents in the majority of conditions require repeated injections [2]. The most frequent complaint during IVI is the varying degrees of pain which causes severe anxiety in one-quarter of patients [4]. In addition to the probability of damage to intraocular structures owing to inadvertent eye movements, the patient’s discomfort and pain associated with the IVI may even cause discontinuation of the treatment [5]. Therefore, it is important to improve the patient’s comfort as much as possible in order to achieve a safer and more durable treatment for best results.

To the best of our knowledge, there is no consensus on the anesthetic agent [6-8], route of anesthetic administration [9-11], pre and post-injection protocols [12-16] for relieving pain, and discomfort related to IVI. Currently, topical anesthesia is the most commonly preferred protocol for IVI due to its safety, cost-effectiveness, and facility [9-11].

The intensity of pain associated with intravitreal injections has been widely investigated in the literature [6,12,17,18], however, there is little and conflicting information about the factors affecting the pain sensation during IVI. Sudden rise in intraocular pressure (IOP), the size of the needle, and the injection technique have been found to be related to pain [19-21]. In the context of these, we aimed to evaluate the effect of anterior chamber paracentesis (ACP) on pain perception and the factors related to pain perception during IVI procedures in this study.

## Materials and methods

**Figure 1:**
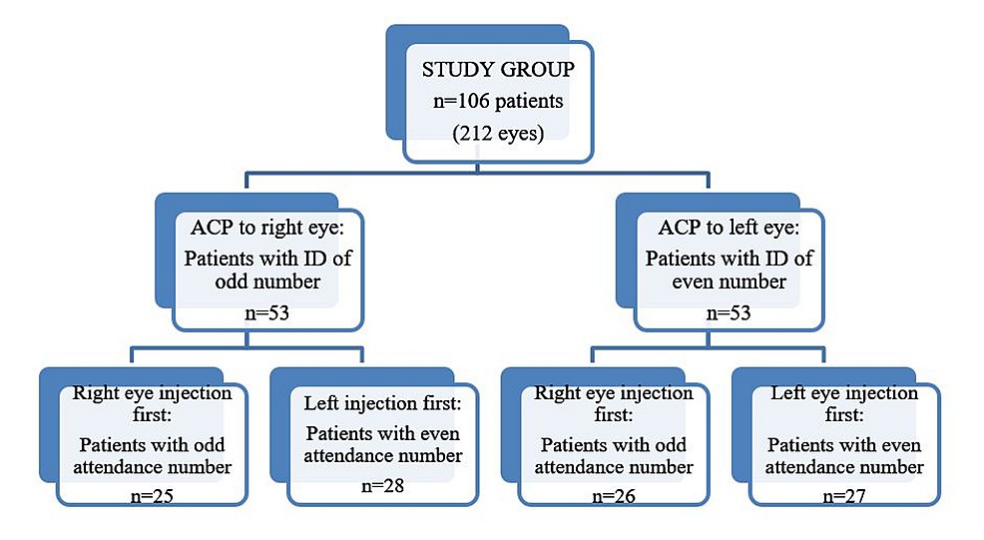
Randomization flow chart ACP: anterior chamber paracentesis

This prospective randomized controlled study conducted in the ophthalmology department of a tertiary university hospital included 212 eyes of 106 patients scheduled for bilateral intravitreal injection of ranibizumab 0.5 mg/0.05 cc (Lucentis®; Novartis Pharma AG, Basel, Switzerland; Genentech, Inc., South San Francisco, CA, USA) under topical anesthesia. Patients’ right and left eyes were randomized to have an IVI with (group 1, n=53) and without ACP (group 2, n=53), according to the personal identification number. The patients with a citizenship identification number ending with an odd number were randomized to group 1, and the patients with an identification number ending with an even number were randomized to group 2. In order to eliminate the possible contribution of the order of the procedure to the perception of pain, patients were randomized again according to the hospital attendance number (Figure 1). IVI with ACP was applied first to the patients with an attendance number ending with an odd number; IVI without ACP was applied first to the patients with an attendance number ending with an even number. This study was conducted in accordance with the Declaration of Helsinki and was given approval by Ordu University Ethics Committee (Approval number: 2018-108). Written informed consents were obtained from all participants. The ocular pain, trigeminal neuralgia, analgesic or sedative use seven days prior to IVI, severe dry eye disease, history of keratopathy or keratitis, history of glaucoma, presence of proliferative retinopathy, active ocular inflammation or infection, nystagmus, allergy to povidone-iodine or ranibizumab or proparacaine hydrochloride, history of ocular surgery and dementia or any other cognitive disease preventing to score VAS were the exclusion criteria. Inclusion criteria were indications for bilateral IVT injection and agreement in taking part in the trial.

A complete medical history was obtained from each patient. Patients underwent a full ophthalmologic examination. Status of the lens, indication for IVI, and laterality were recorded. Axial length (AL) and anterior chamber depth (ACD) were measured with a combined biometric pachymeter (PacScan 300AP Digital Biometric Ruler; SonoMed, Lake Success, NY, USA).

Anesthesia administration

All patients received topical anesthesia with 0.5% proparacaine hydrochloride (Alcaine 0.5%, Alcon Pharmaceuticals, Puurs, Belgium) drops applied to the ocular surface up to two times five minutes apart before the injection.

Injection technique

All of the injections were performed by a single right-handed surgeon while the patient was in the supine position. Following cleaning the area with 10% povidone-iodine and placement of a single-use sterile adhesive surgical ocular drape, a disposable lid speculum was inserted. Povidone-iodine 5% was instilled in the conjunctival cul-de-sacs three minutes before the injection. After the prophylactic slow free-flow ACP with a 1 ml insulin syringe needle (26-gauge), the injection was performed in two seconds via a 30-gauge needle perpendicularly in the inferior temporal quadrant, 3.5 mm from the limbus in aphakic/pseudophakic patients and 4.0 mm in phakic patients with the guidance of a sterile caliper. A sterile cotton-tipped applicator was used after removing the needle for a gentle pressure to prevent reflux. Reflux and subconjunctival hemorrhage (SCH) were recorded if present. Following hand-motion control, a drop of antibiotic was instilled. The same procedure was executed in the contralateral eye in the same order except for anterior chamber ACP. Patients were invited to rest for half an hour for IOP measurement at 5 and 30 minutes. The patients were instructed to self-administer 0.5% moxifloxacin (Vigamox; Alcon Laboratories, Inc., Fort Worth, TX, USA) four times a day during the following five days.

Assessment of pain perception

After the operation, the patients were asked to grade the pain they experienced by a Visual Analogue Scale (VAS) from 0 (no pain) to 10 (unbearable pain).

Statistical analysis

Data analyses were performed by using SPSS for Windows, version 22.0 (SPSS Inc., Chicago, IL, USA). Whether the distributions of continuous variables were normal or not was determined by the Kolmogorov Smirnov test. Levene test was used for the evaluation of homogeneity of variances. Data are expressed as mean±standard deviation for continuous variables and number (percentage) for categorical variables. The Wilcoxon test was applied for independent data. Categorical dependent variables were evaluated with the McNemar test. It was evaluated the degree of relationship between variables with Pearson or Spearman correlation analysis. A p-value < 0.05 was accepted as a significant level on all statistical analyses.

## Results

A total of 212 eyes of 106 patients who met the inclusion criteria were enrolled and randomized to have an IVI with (group 1) and without anterior chamber ACP (group 2). The study group consisted of 55 females (51.9%) and 51 males (48.1%) and the mean age was 67.20±11.72 (mean ± SD) with a median of 68 years. The indication for IVI was bilateral diabetic macular edema in 48 (45.3%) and neovascular age-related macular degeneration in 58 (54.7%) patients.

Ninety-eight (46.2%) eyes were IVI-naive. The mean number of injections in eyes with a history of previous injections in groups 1 and 2 were 3.44±1.92 and 3.29±2.02, respectively (p:0.703).

Group 1 consisted of 75 (70.7%) phakic and 31 (29.2%) pseudophakic eyes whereas group 2 consisted of 76 (71.7%) phakic and 30 (28.3%) pseudophakic eyes at the time of IVI (p:1.00).

Mean ACD and AL were measured as 3.23±0.44 mm and 23.31±1.36 mm in group 1, respectively, and mean ACD and AL were measured as 3.24±0.42 mm and 23.43±1.34 mm in group 2, respectively (p:0.008, p<0.001).

Two (1.9%) eyes in group 1 and 17 (16%) eyes in group 2 experienced reflux (p<0.001) and SCH was detected in two (1.9%) eyes in group 1 and seven (6.6%) eyes in group 2 (p:0.059). Characteristics of the patients for the groups are shown in Table 1.

**Table 1:** Characteristics of the patients Data are expressed as mean ± SD for continuous variables and number (percentage) for categorical variables. *Wilcoxon test, ^β^Mc-Nemar test. Statistically significant p-values are in bold. IVI: intravitreal injection, ACD: anterior chamber depth, AL: axial length, SCH: subconjunctival hemorrhage, Pre-IOP: pre-injection intraocular pressure, Post-IOP 5 min: post-injection 5 minutes intraocular pressure, Post-IOP 30 min: post-injection 30 minutes intraocular pressure.

	Group 1		Group 2		P-value
Prior IVI^β^	59	(55.7)	55	(51.9)	0.344
Number of IVI^*^	3.44	±192	3.29	±2.02	0.703
Pseudophakia^β^	31	(29.2)	30	(28.3)	1.000
ACD^*^	3.23	±0.44	3.24	±0.42	0.008
AL^*^	23.31	±1.36	23.43	±1.34	<0.001
Pre-IOP^*^	13.30	±2.85	13.15	±2.45	0.272
Post-IOP 5 min^*^	12.21	±2.66	24.15	±6.82	<0.001
Post-IOP 30 min^*^	12.99	±2.66	18.89	±3.06	<0.001
Pain^*^	0	(6.00)	1.00	(8.00)	<0.001
Reflux^β^	2	(1.9)	17	(16)	<0.001
SCH^β^	2	(1.9)	7	(6.6)	0.059

Mean pre-injection IOP values in groups 1 and 2 were measured as 13.30±2.85 mmHg and 13.15±2.45 mmHg, respectively (p:0.272). Mean IOP values measured five minutes post-injection were 12.21±2.66 mmHg and 24.15±6.82 mmHg in groups 1 and 2, respectively (p<0.001). In addition, mean IOP values measured 30 minutes post-injection were 12.99±2.66 mmHg and 18.89±3.06 mmHg in groups 1 and 2, respectively (p<0.001).

Pre-injection IOP values and 30 minutes post-injection IOP values for group 1 were not statistically different, in fact, there was a significant decrease in IOP values at five minutes post-injection IOP values compared to pre-injection IOP (p<0.001). Whereas there was a significant rise in post-injection IOP values at 5 minutes and 30 minutes compared to pre-injection IOP values for group 2 (p<0.001) with a median increase of 9.5 mmHg (Table 2).

**Table 2:** Median pre-injection IOP and change in IOP at post-injection 5 and 30 minutes Data are expressed as median (range) for continuous variables. *Friedman test, ^β^Chi-square. Significant differences were found between; p1: pre vs post 5 min, p2: pre vs post 30 min. Statistically significant p-values are in bold. Pre-IOP: pre-injection intraocular pressure, Post-IOP 5 min: post-injection five minutes intraocular pressure, Post-IOP 30 min: post-injection 30 minutes intraocular pressure.

	Pre-IOP	Post-IOP 5 Min	Post-IOP 30 Min	P-value	Post hoc test
Group 1	13 (13)	12 (10)	13 (12)	<0.001	p1<0.001; p2:0.320
Group 2	13 (12)	22.5 (40)	18.5 (20)	<0.001	p1<0.001; p2<0.001

The mean VAS score for groups 1 and 2 was recorded as 0.51±1.00 and 1.32 ±1.50, respectively (p<0.001). Reflux was observed significantly more often in group 2 when compared to group 1 (p<0.001).

The correlations of VAS score with the age, gender, indication for IVI, presence of DM, history of previous IVI, number of previous injections, lens status, pre-injection IOP, AL, ACD values, presence of reflux, and SCH for both groups are shown in Table 3. VAS score was correlated with a history of previous IVI, preinjection IOP in both group 1 (p:0.001, p:0.007) and group 2 (p:0.046, p:0.009), whereas inversely correlated with the presence of reflux in both groups (p:0.018; p:0.038) and ACD (p:0.032) in group 2.

**Table 3:** Correlation analysis for pain The degree of relationship between variables was evaluated with Spearman’s rho correlation and point biserial correlation analysis. IVI: intravitreal injection, ACD: anterior chamber depth, AL: axial Length, SCH: subconjunctival hemorrhage, Pre-IOP: pre-injection intraocular pressure.

		VAS score group 1	VAS score group 2
Age	r	−0.065	−0.072
	p	0.511	0.466
Gender	r	0.056	0.025
	p	0.571	0.800
Indication	r	−0.113	−0.246
	p	0.249	0.011
Prior IVI	r	0.321	0.194
	p	0.001	0.046
Number of IVI	r	0.103	0.257
	p	0.438	0.058
Lens status	r	−0.044	0.139
	p	0.658	0.157
ACD	r	−0.185	−0.208
	p	0.058	0.032
AL	r	−0.184	−0.156
	p	0.060	0.110
PRE-IOP	r	0.261	0.254
	p	0.007	0.009
REFLUX	r	−0.229	−0.202
	p	0.018	0.038
SCH	r	0.094	0.073
	p	0.338	0.455

No ocular or systemic adverse events owing to paracentesis or IVI were encountered in both groups except SCH.

## Discussion

VEGF-targetted intravitreal drug injections in the treatment of retinal diseases, where overexpression of VEGF leads to vascular leakage and neovascularization, constitute one of the most frequently practiced procedures in ophthalmology. Although therapies with anti-VEGF agents were shown to be effective and safe, frequent injection need warrants long-term follow-up and compliance of patients. The most frequent complaint during IVI is the varying degrees of pain which cause severe anxiety [4] and even discontinuation of the treatment [5]. We, therefore, aimed to evaluate the effect of ACP on pain perception and assess the factors related to pain perception during IVI procedures.

Demographic factors such as age and gender do not seem to affect the perception of pain during IVI [12,18,22], as well as the side of the injected eye and or indication of treatment [6], in concordance with the results of this study. However, Haas et al. reported that older patients, female patients, and patients having previous IVI had higher pain scores [21]. Massamba et al. suggested that injections of the left eye and temporal superior quadrant yielded significantly greater pain scores [22]. In this study, the history of multiple IVI was found to be associated with greater pain perception in our patients in both groups 1 and 2 resembling the results of Haas et al. [21] and in contrast to Rifkin and Schaal [7] which may reflect the scleral wound healing with the shrinkage of the collagen and increased rigidity.

The impact of needle size on pain perception during IVI has been investigated by various authors. While some studies reported lower pain with 30-gauge needle injections than 27-gauge needles [19,23], others found no significant difference in pain scores when compared to the 27- and 30-gauge needles [7,20,21]. However, many ophthalmologists favor smaller-sized needles [24], owing to the belief that it induces less pain. So, in this study, all of the injections were applied via a 30-gauge needle in the same quadrant. In addition, the right and left eyes of the same patients were randomized to eliminate the interindividual variability in pain perception.

In this study, ocular biometrics were found to be significantly related to pain perception in accordance with the report by Gismondi et al. [25]. Shorter ACD in group 2 was associated with greater pain which may be explained by greater scleral thickness and rigidity and decreased outflow capacity. AL and ACD values had no impact on pain perception in group 1 patients where ACP was performed, despite the significantly lower values compared to group 2.

It is known that depending on the volume effect, transient but significant IOP rise occurs immediately after the intravitreal anti-VEGF injection. Bracha et al. demonstrated an average of 46 mmHg IOP rise which returns to baseline within one hour in healthy eyes [26]. This sudden rise in IOP may contribute to pain sensation. In this prospective randomized study, the VAS score was significantly lower in the paracentesis group (group 1) which may reflect the absence of postinjection hyperacute IOP rise. Post-injection IOP measurements both at 5 minutes and 30 minutes were significantly higher in group 2 than in group 1. Eyes in group 2 experienced an acute IOP rise of median 9.5 mmHg five minutes after injections, but no rise in group 1. Moreover, patients in group 1 experienced lower IOPs than baseline in measurements five minutes after the injections. Risk factors for acute IOP rise were defined by Bracha et al. as the absence of subconjunctival reflux, prior history of glaucoma, smaller vitreous volume manifested by a short AL, and possibly volume of the injected drug [26]. Knip and Välimäki demonstrated the effectiveness of an ACP over an IOP spike (postinjection IOP was 15.3 mmHg versus 47.1 mmHg in the paracentesis and control group, respectively) [27]. It was suggested that ACP may prevent sustained IOP elevation [28] and also retinal nerve fiber loss [29], however, definitive evidence is lacking.

Although there is no consensus regarding its necessity, an ACP seems to offer a more effective, however, riskier method for the prevention of acute ocular hypertension following IVI [26]. Despite we did not encounter any adverse event owing to the ACP in this study, there are reported complications in 0.7% of patients such as inadvertent injection of sterile air into the anterior chamber, anterior lens capsule laceration, allergic reaction to povidone-iodine in a large series as well as hyphema, lens damage, and infection [30].

The main limitation of the current study was the small sample size of a single center. Further large-scale studies are needed to achieve patient comfort and compliance to the treatment of IVI therapies.

## Conclusions

Intravitreal administration of pharmaceutical agents offered an effective route for reaching high concentrations at the target tissue. However, the regimens for retinal diseases treated with anti-VEGFs require more than a single injection. These therapies with frequent injections of anti-VEGF agents warrant long-term follow-up and compliance of patients. Pain may interfere with compliance with treatment. Although it possesses some potential bothersome risks such as infection and iatrogenic lens damage, ACP offers a comfortable, effective, and less painful step in IVI by preventing an acute rise in IOP experienced immediately after IVI, especially in patients with small anterior chambers, small vitreous volumes, history of previous IVIs and in patients at risk of optic disc hypoperfusion with advanced glaucomatous optic neuropathy.
